# Successful Organ Donation After Yew Intoxication

**DOI:** 10.3389/ti.2025.13962

**Published:** 2025-03-13

**Authors:** P. Appelt, A. Stuerzebecher, L. Weidhase, S. Ziganshyna

**Affiliations:** ^1^ Leipzig University, Faculty of Medicine, Leipzig, Germany; ^2^ Interdisciplinary Medical Intensive Care, University of Leipzig Medical Center, Leipzig, Germany; ^3^ Poisons Information Centre Erfurt, Erfurt, Germany; ^4^ Organ Donation Coordinator Unit, University of Leipzig Medical Center, Leipzig, Germany

**Keywords:** ECLS, yew intoxication, organ donation, organ protection, intensive care medicine

Dear Editors,

We would like to report on a case of a successful organ donation under ongoing extracorporeal life support following a yew intoxication.

The European yew (*Taxus baccata*) is as an ornamental conifer widespread throughout Europe. The poisonousness of yews has been known since ancient times and can lead to life threatening intoxications. All parts of the plant, with exception of the red aril, are poisonous. Measured by its cardiotoxic effect, Taxin B is the most important of the alkaloids contained in yews called taxanes. An ingestion of about 3–6.5 mg/kg bodyweight Taxine B is described as potentially lethal for humans [1]. In central Germany there is approximately one severe intoxication per year; most intoxications occur with suicidal intent in young adults [2, 3].

In Germany, brain death (BD) is a necessary condition for organ donation. The second frequent cause of BD is the hypoxic-ischemic encephalopathy (HIE) following cardiac arrest [4]. Life threatening yew intoxications are rare [2], but can lead to cardiac arrest and so to HIE.

A few years ago, we treated a middle-aged patient with a lethal yew intoxication. For ethical reasons, age and gender can not be specified. The medical history included paranoid schizophrenia, recurrent depressive episodes, and several suicide attempts. The patient ingested around 50 crushed yew needles with suicidal intent. Later, regretting the ingestion, the patient sought medical assistance.

Emergency services were called to the location and found a hemodynamically stable patient, already showing a broad complex tachycardia on the ECG. Arriving at a primary care hospital, the patient showed a hemodynamic relevant ventricular tachycardia, which deteriorated into an asystole following electrical cardioversion. After a brief, successful cardiopulmonary resuscitation (CPR), an esophagogastroduodenoscopy (EGD) with application of activated charcoal was performed, and the patient was transferred to a higher-level care facility. As the broad complex tachycardia persisted and a cardiogenic shock developed, a multi-faceted therapy with administration of high dose catecholamines, application of lidocaine, sodium bicarbonate, and digitalis antidote was established. Additionally, a continuous hemodialysis with hemadsorption was applied. Under this therapy the ECG rhythm stabilized temporally before suddenly another cardiac arrest with an asystole occurred. A pacemaker could not be implanted. This time, CPR was prolonged and, as a return of spontaneous circulation (ROSC) did not occur, an extracorporeal life support system (ECLS) was established successfully 1 hour after the start of CPR. The treatment, after ROSC, addressed a post-cardiac arrest syndrome, with persistent hemodynamic instability, renal failure, and a prolonged metabolic acidosis. During therapy, a lack of wake-up reaction and a loss of brainstem reflexes were observed. As a CT scan of the head showed a pronounced cerebral edema indicating severe HIE, an isoelectric EEG was derived. All criteria of BD were fulfilled. In accordance with the patient’s wishes, as expressed by the relatives, the patient was evaluated as an organ donor.

We continued intensive care therapy for 3 days, fully aware of the yew intoxication, to ensure that no toxins remained in the blood. Blood levels of 3,5-dimethoxyphenole and other taxanes, including Taxin B, measured by liquid chromatography–mass spectrometry (Triple Quad 5500+, Sciex, Framingham, USA) were taken on days 1, 2, and 3. It was only on day 3 that no toxins were detected, and therefore BD diagnostics were initiated. Organ-protective intensive care, including ECLS, was maintained until the irreversible BD was confirmed. Subsequently, the liver and both kidneys were successfully donated. The organ quality was assessed as good. Histologic examination of the liver showed a microvesicular fibrosis and this organ was rejected for transplantation. The kidneys were successfully transplanted and showed a good one-year-organ function (Table 1).

**TABLE 1:** Results of the organ donation.

Organ	Time of explanation		1-year after transplantation			
	Visual organ quality	Transplantation	Creatinine [mmol/L]	Urea [mmol/L]	GFR [mL/min/1,73 m^2^]	Summarized organ quality^a^
Left Kidney	Good	Yes	166	5,6	38	Good

Right Kidney	Good	Yes	142	6,3	43	Good

Liver	Good	No Histology: microvesicular steatosis	-	-	-	-

^a^ Assessment through the ministering transplantation centers.

The ingestion of 50 yew needles represents as a potential lethal dose [1]. By inhibition of myocardial sodium and calcium channels, Taxin B induces a transient myocardial channelopathy [5, 6]. Clinically, patients present with malignant cardiac arrhythmias and cardiogenic shock. Patients with a potentially fatal poisoning should immediately be admitted to a tertiary care center with access to a full range of treatments. Otherwise, sufficient therapy may be delayed. Many therapy options are described in the literature, very few of them with evidence. Gastrointestinal decontamination seems to be an useful therapy option, because Taxin B induces a prolonged gastric passage. Therefore, the repetitive administration of activated charcoal is recommended [7]. An EGD is an individual measurement and not generally recommended. For potential lethal yew intoxications both well-established therapies should be applied.

There are no options for secondary toxin elimination: Due to the large size of Taxin B molecules (534 kDa) neither hemodialysis nor hemadsorption filters are able to eliminate yew alkaloids. Symptomatic therapy options for yew intoxication are limited as well. With a half-life of 11–13 h [8], the effect of yew toxins can be expected to last for 2 days. Symptomatic therapy should bridge this time. This is consistent with the toxicological measurements in the presented case. There is some evidence of ineffectiveness of sodium bicarbonate, Lidocaine should be avoided due to its similar effect to Taxine B. The use of pacemakers or an electrical cardioversion is uncertain, because of the underlying myocardial channelopathy. A reported cross-reactivity of yew alkaloids with digitalis antidote was the rationale of its use [9]. Best data exists for ECLS [9], despite the fact that it is a procedure prone to complications.

The rationale for the use of digoxin immune fab is the potential cross-reactivity of the taxane constituents in the yew plant with the digoxin-specific antibody fragments.

Taxin B has a cardioselective effect [6], so that organ damage in severe yew intoxications does not primary result from the toxin but secondarily by the induced cardiogenic shock and cardiac arrest. The observed microvesicular steatosis of the liver is caused by an impaired mitochondrial beta-oxidation. There is no described effect of taxanes on mitochondrial functions and thus considered as the impact of hepatic hypoxia due to a prolonged cardiogenic shock and CPR time. At the time of the declaration of BD, taxanes could not be detected. Postmortem histological analysis of the myocardium showed no signs of inflammation or other pathologies. No long-term effects of taxanes are known, and none were observed in the transplanted organs.

In conclusion, an organ donation after a lethal yew poisoning is possible and leads to good transplantation outcomes. Therefore, ECLS is not only the option with most evidence in therapy of yew intoxications, it also provides an option to optimize organ protective intensive care therapy towards organ donation.

## Data Availability

The original contributions presented in the study are included in the article/supplementary material, further inquiries can be directed to the corresponding author.
